# Criteria for Preliminary Risk Assessment of Brownfield Site: An International Survey of Experts

**DOI:** 10.1007/s00267-022-01684-x

**Published:** 2022-08-04

**Authors:** Charf Mahammedi, Lamine Mahdjoubi, Colin Booth, Russell Bowman, Talib E. Butt

**Affiliations:** 1 Brownfield Research and Innovation Centre (BRIC), University of Wolverhampton, Wolverhampton, WV10 0JP UK; 2 Centre for Architecture and Built Environment Research (CABER), University of the West of England, Bristol, BS16 1QY UK; 3 Soil and Structures Ltd, Glasgow, UK; 4 Faculty of Engineering and Environment, Northumbria University, Newcastle-upon-Tyne, NE1 8ST UK

**Keywords:** Brownfield sites, Professional perception, Preliminary risk assessment, Decision-making, Site investigation, Pollutant linkage model

## Abstract

Comprehensive risk assessment of brownfield sites requires a broad range of knowledge and multi-disciplinary expertise. Whilst the identification of criteria requirements for preliminary risk assessment has received some attention, there appears to be no studies that have specifically examined professional perspectives relating to these requirements. Yet, variations in professional practitioners’ assessments may have significant consequences for the assessment of risks, and how the criteria are imparted to stakeholders. This study aims to identify the criteria requirements for preliminary risk assessment, using the pollutant linkage model (Source–Pathway–Receptor), and explores cross-disciplinary professional perspectives related to these requirements. To this end, this study commenced with a systematic review to identify various criteria streams required for the preliminary risk assessment of brownfield sites. Thereafter, a questionnaire survey was design and shared with brownfield site professionals. Quantitative analysis of the survey responses (*n* = 76) reveals disciplines have markedly different priorities relating to the same hazard. For instance, geophysicists, geochemists, and hydrologists do not raise concerns regarding ground movement that can result from the removal of storage and tanks, whilst the same hazard was considered as having a high importance by other professions (such as geologists and geotechnical engineers). This example, amongst others revealed in the study, underpins potential issues and implications for various stakeholders compiling and/or using preliminary risk assessment criteria. This study clarifies both the key criteria requirements for the preliminary risk assessment of brownfield sites, as well as the importance of recognising how variation in professionals’ perceptions plays in the risk assessment process. Although, specialist knowledge is essential for brownfield site investigation, so is the maintaining a broad-based view of other experts coming from different backgrounds, as this renders holistic risk assessment insights.

## Introduction

Preliminary risk assessment has been more common in recent years as one of the critical stages for brownfield management, particularly when soil or groundwater contamination is involved (Butt et al. 2020; Mahammedi et al. 2020a; Cushman et al. 2001). This phase of risk assessment aims to establish whether there are any potentially unacceptable risks with the site, whether any further information is likely to be needed to complete this stage or whether the site needs to be kept under review (Environment Agency 2016). The assessment process usually involves the analysis of substantial and wide ranging information to identify potential or existing constraints affecting the site or that could affect the site in the future (Martin and Toll 2006).

The main methodologies for performing risk assessments are provided by the US Environmental Protection Agency (U.S. EPA 2019, 2014, 1989, 2001, 1997), the UK Environment Agency (EA) (Environment Agency 2008; DEFRA; Environmental Agency 2004; Environment Agency 2009, 2015, 2017), and the Canadian Council of Ministers for the Environment (CCME) (Health Canada 2010). According to Cushman et al. (2001), there are three main types of risk assessment used for addressing brownfield related issues: a human health risk assessment, an environmental risk assessment and building structures. A human health risk assessment evaluates the risks associated with human exposures to contamination. An environmental risk assessment evaluates the risks associated with flora and (or) fauna exposures to contamination. Building structures risk assessment, which is less prominent than the first two, but no less important, assesses the risks posed to building structures (i.e. permeation and (or) degradation of underground utilities, sewers, building foundations, etc.) due to contact with contamination.

A systematic review and analysis of the available risk assessment literature for brownfield and contaminated sites was conducted by Mahammedi et al. (2020a), who identified 31 tools and holistically classified them in terms of risk assessment stages, and types of harms, hazards, pathways and receptors. The results show that risk analysis tools for contaminated sites are detailed, complex, time consuming, effort-intensive and costly for preliminary assessment. It establishes the escalating need of preliminary risk assessment tools which are appropriately detailed, nothing more, nothing less. Another review was published by the European Environment Agency (EEA, 2004), where a number of documented international methodologies are listed and analysed. The approaches reviewed are mostly used to rank potential contaminated sites based on existing data in order to develop priority action plans related to detailed site survey and remediation. The reviewed methodologies follow a qualitative method to assess the risks raised by potential contaminated sites. They define the three components of a risk assessment model (i.e. source, pathway and receptor) in terms of scores for assessing related risks, instead of absolute estimates of health/environmental impacts (Zabeo et al. 2011; Pizzol et al. 2011). Prioritisation methodologies, including the Multi–Criteria Decision-Making (MCDM) method, have been proposed in a range of brownfield regeneration process (Linkov et al. 2020; Cinelli et al. 2021), including the application of AHP and Fuzzy AHP for forest conservation (Wolfslehner et al. 2005; Laxmi et al. 2012), landfill site selection (Wang et al. 2009; Donevska et al. 2012), site selection (Chen 2006; Vahidnia et al. 2009), remediation techniques (Linkov et al. 2004; Promentilla et al. 2008), and VAHP for potential hazards associated with brownfield sites ((Mahammedi et al. 2021).

Inadequate site assessment may expose investigation personnel, and the general public, to unnecessary and unacceptable risks. These can even lead to more extensive or intractable contamination problems than those that previously existed on a site (Harris and Herbert 1994; Mahammedi et al. 2020b). Land acquisition without appropriate investigation can result in the developer incurring financial and legal liabilities. For instance, Shepherd (2020) reported a case study where a buyers bought a houses in Bradford, UK without preliminary risk assessment. After acquisition, the houses were found worthless because the estate backs onto what used to be a landfill site. Despite it being inactive for over four decades, the council says it still releases toxic methane gas. This meant the scheme design adversely impacted the project profit. In another example, cases of ill health were recorded affecting some residents in the former mining area in Midlothian, Scotland. Investigations revealed residents were suffering from exposure to carbon dioxide (CO_2_) released from historical coal mines beneath their homes. Demolition of 64 homes was the only option to prevent the possibility of further leaks of carbon dioxide into these homes over the longer term (BBC 2014). Both incidents serve as a stark reminder of the potential jeopardies involved with reusing of brownfield sites.

Successful investigation of brownfield sites typically requires multi-disciplinary expertise and a multi-staged approach, as well as multi-agency regulation to analyse the immense volume of information needed to make a complete risk assessment of a site (Nathanail 2009, 2013; Marsili 2016). Risk assessment is highly complex and requires information from many disciplines, taking into consideration the range of contexts in which decision have to be made, including complying with industry standards, relevant legislative frameworks, health and safety issues, accounting for total operating costs and benefits, and addressing issues of environmental impacts, sustainability, protection of other resources, and importantly the prevention of further and/or future contamination (Bello-Dambatta 2010). One of the key challenges is an enhanced awareness of the varying priorities and competencies that other professionals working on brownfield sites have and how these might be reconciled for more effective risk assessment. Amongst the difficulties facing brownfield site assessors is the quantity of potential risks on the development of brownfield sites that are often far from what assessors can expect to identify (Kovalick and Montgomery 2017). This may increase misunderstanding and communication issues between various stakeholders.

The risk assessment process for sites covers a range of knowledge branches such as the environment, geology, hydrology, geotechnics, chemistry, and alike. Consequently, the process requires engagement from and with a wide range of experts from different backgrounds. According to Nathanail (2009), engineering geology has an essential role to play in ensuring that risk assessments are applied, appraised, and implemented in ground investigations. For example, the fate and transport of contaminants is a function of engineering geological parameters (solubility, volatilisation, etc.) and the properties of the ground they are in (clay content, pH, organic matter content, etc.). In addition, Jefferis (2010) indicated that geotechnical engineers should be encouraged to pro-actively minimise the risk of future contaminated land. They should be prepared to use their accumulated experience of the behaviour of chemicals in the ground and groundwater environments to raise concerns about the widespread use or use without sufficient protection of chemicals that are manifestly dangerous to the environment.

There is a need for more inclusive criteria coming from the perspective of various professional practitioners in view of their different backgrounds; thereby, enabling a more holistic and complete identification of hazards (with their diverse implications) for a given brownfield site. Having prior knowledge about the typical information that should be gathered to identify the three components of the pollutant linkage model (Source–Pathway–Receptor) reduces the risk of encountering unforeseen hazards and decreases the unnecessary cost of the site investigation. The source of hazards in brownfield sites are investigated by Vik and Bardos (2003), Environment Agency (2004, 2008), Harrison (2015), and Mahammedi (2021) it was concluded that the main source of hazards is the chemical and biological contamination arising from past industrial use, which may present a major threat to different human health and built environment. Furthermore, Leach and Goodger (1991), Charles et al. (2002), Charles (2005), Wilson et al. (2007) investigate physical hazards including ground movement and obstructions (i.e. buried foundations, underground services, old tanks etc.). Pathway identifies how hazards were released from the source into the environment (Butt et al. 2016). The pre-exposure is mainly subjected to investigate the impact of site conditions including site geology, hydrology and topography on the fate and transport of contaminants.

From the perspective of brownfield sites, preliminary risk assessment involves collecting enough reliable and accurate criteria to identify the three component of pollutant linkage model. For the three components, no evidence has been found of particular studies that can help to identify the required criteria to establish the pollutant linkage model more holistically and categorically. This study is not about the preliminary risk assessment itself, as such which is hazard identification and hazard assessment (DEFRA and Environment Agency 2004; AECOM Infrastructure and Environment UK Ltd 2017). The study is rather about identifying and characterising/categorising the types of data and information which are fundamentally required to form the basis of preliminary risk assessment. The study signifies such data and information without which preliminary risk assessment cannot be conducted in the first place. The aim of this study is to identify the risk assessment criteria of brownfield site at early stage of risk assessment based on pollutant linkage model.

## Research Design and Methodology

**Fig. 1:**
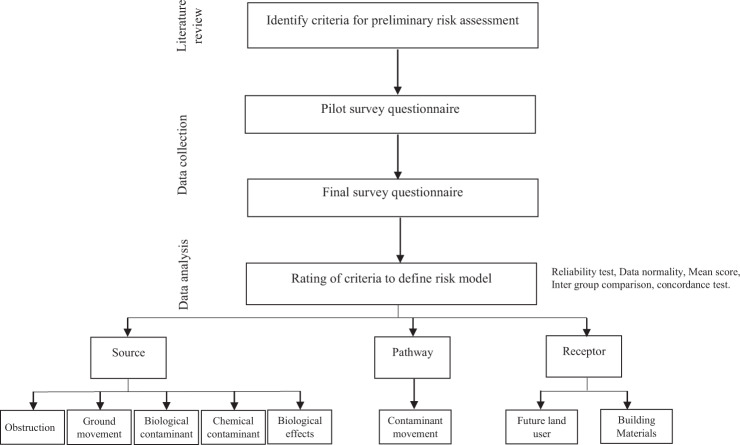
Overall research design

This study adopts a quantitative research strategy; whereby, after a comprehensive review of brownfield site literature, a questionnaire survey was used for the collection of empirical data. An overview of the process adopted for this study is detailed below (Fig. 1), which shows a four-stage process. Stage one identifies the criteria for preliminary risk assessment based on existing literature. Stage two uses a questionnaire administered to disciplinary experts to validate the literature findings. Finally, stage three comprises statistical analysis of the survey data using the SPSS 26.0 statistical package to enable conclusions to be drawn out.

### Identification of Criteria for Preliminary Risk Assessment of Brownfield Sites

In order to identify the key criteria for preliminary risk assessment, it was decided to screen the literature. This review was conducted on academic and professional databases, plus grey literature. The academic database included: Scopus, American Society of Civil Engineers (ASCE), Institution of Civil Engineers (ICE) virtual library and other relevant literature including government guidance and technical reports. The search words were a combination of “Preliminary criteria”, “Hazard assessment”, “Hazard identification”, “Contaminated sites”, “Brownfield sites”, “Site investigation”, “Site appraisal” and “Site report”. They were selected for their relevance to preliminary risk assessment of brownfield sites and returned relevant literature from the majority of main journal and conference publications.

After removing duplicates subsequent exclusion rounds were completed through reading of the titles, then the abstract and finally the full articles. The following suitability criteria were adopted: (i) relevant literature that does concern preliminary assessment and brownfield sites, (ii) adequate quality. The review findings are presented in Table 1.

**Table 1:** Criteria needs for preliminary risk assessment

Reference	History of the site	Surrounding areas	Buildings and other structures	Underground services	Storage of materials and old tanks	Site geology	Previous mining activities	Presence of radon	Invasive species	Made ground	Site hydrogeology/hydrology	Site topography	Receptors
(New Jersey Department of Environmental Protection (NJDEP) 2019)	✓	✓	✓	✓	✓	✓					✓		
(Nikolaidis 2018)	✓					✓					✓		
(Department of environmental conservation 2017)	✓	✓	✓		✓						✓	✓	
(Suthersan et al. 2016)	✓					✓					✓		
(Nathanail et al. 2011)	✓	✓	✓	✓	✓	✓					✓		
(Büyüker 2009)	✓					✓					✓		✓
(Environment Agency 2008)	✓	✓	✓	✓	✓	✓	✓	✓	✓	✓	✓	✓	✓
(Department of Toxic Substances Control (DTSC) 2008)	✓	✓				✓					✓	✓	✓
(Martin and Toll 2006)	✓	✓	✓	✓	✓	✓					✓	✓	✓
(DEFRA; Environment Agency 2004)	✓	✓	✓	✓	✓	✓						✓	
(Regens et al. 2002)	✓	✓				✓					✓	✓	✓
(McMahon et al. 2001)	✓					✓					✓		✓

### Survey Design, Sample Recruitment and Data Collection

A questionnaire was developed using the online survey tool Qualtrics. To determine the convenient target groups for the online survey, the questionnaire adopted the purposeful sampling method. Also called judgement sampling, this technique is a non-random procedure, in which the researcher relies on his or her judgement when selecting members of the subjects to participate in the study (Saunders et al. 2019). Purposive sampling is employed because the investigator is looking for strong information in a certain area of expertise and wants to learn more about the subject. Therefore, the survey is limited to companies with brownfield management experience, working in the UK and North America. These were selected from the main brownfield groups on LinkedIn. These groups included Brownfield Briefing (739 members), Property and Real Estate Development, Town Planning, Design, Funding and Construction Solution (5825 members), CABERNET—Europe’s brownfield regeneration network (member 548), UK Brownfield Investigation Assessment and Remediation (812 members), Construction Industry Research and Information Association (CIRIA) (3000 members), Florida Land Development News (343 members). The survey was divided into two main sections. The first section requested demographic information about the participant’s background and years of experience. The second section adopted a five-point Likert scale (Table 2) to rate the criteria with a five-point rating scale (1 = not important, 2 = less important, 3 = neutral, 4 = important, and 5 = very important). It comprises 49 questions across 7 sub-sections, covering: (i) obstruction hazards (nine questions); (ii) ground movement (nine questions); (iii) chemical contaminants (nine questions); (iv) biological hazards (nine questions); (v) biodegradable effects hazards (nine questions); (vi) contaminant movement (four questions); and (vii) receptor (two questions). In this study, Likert items are considered an interval level data with distance between the points. Therefore, the data analysis decision for Likert scale items can use the mean to measure the central tendency. It important to mention that three academic professionals piloted the online survey before it was accepted as a final survey, focusing on question construction, this ensured that the questionnaire was meaningful and easy to follow.

**Table 2:** Five-point Likert scales used in the survey questionnaires (Pimentel 2010)

Likert scale	Interval	Linguistic terms
1	1.00–1.79	Not at all important
2	1.80–2.59	Slightly important
3	2.60–3.39	Moderately important
4	3.40–4.19	Very important
5	4.20–5.00	Extremely important

Participants are asked to read and understand the participant consent and participant information sheet, and then if they are interested, they could proceed via an attached link to the survey. The survey was left open for 4 months to collect the highest number of responses. Moreover, ethics and moral standards are integral to research studies. Therefore, all participants were informed their involvement was voluntary and their decision to return their questionnaire would be deemed as their consent to take part in the survey. As their responses would be anonymous, participants were invited to create their own unique identification code in case they wished to withdraw up to 2 weeks after the completed survey had been returned. The study was conducted in accordance with the ethics regulations at the University of the West of England (UWE), Bristol.

## Data Analysis

The data collected from the survey was analysed using various statistical analysis methods, which are described in this section.

### Reliability Test–Cronbach’s Alpha

Cronbach’s alpha test remains one of the most popular methods for assessing the reliability, or internal consistency, of a set of scale or test items. It is computed by correlating the score for each scale item with the total score for each observation and then comparing that to the variance for all individual item scores. Data is said to have high reliability if it produces similar results under consistent conditions. The Cronbach’s alpha coefficient value ranges from 0 to 1, and the higher the value, the more reliable is the adopted scale of measurement. Tavakol and Dennick (2011) argued that, if the alpha value is above 0.70, it indicates an excellent internal consistency within the data. Using SPSS, the Cronbach’s alpha coefficient value could be calculated as following (Darko 2019)

$$\alpha \,=\, \frac{{N \,\cdot\, \overline c }}{{\overline v \,+\, \left( {N \,-\, 1} \right) \,\cdot\, \overline c }}$$

Where: N = the number of items. $\overline c$ = average covariance between item–pairs. $\overline v$ = average variance

In this study, Cronbach’s alpha coefficient test was used to assess the reliabilities of the five-point rating scales used to capture the survey responses.

### Mean Score

The mean score of the importance of the criteria is calculated using the following formula:

$$B_i \,=\, \frac{{\mathop {\sum}\nolimits_{j \,=\, 1}^n {\alpha _{ij}} }}{n}$$

Where *n*: the total number of participants; *α*_*ij*_: the importance of the criteria *i* rated by the participant *j*; and *B*_*i*_: the mean score of the importance of the criteria *i*. The SPSS statistical software was used to calculate the mean score for the criteria, and for ranking the criteria. For research rigour, only criteria with mean scores higher than 3.40 was important. This approach was adopted from Pimentel (2010) and does not only determine the necessary criteria to identify pollutant linkage model, but also helps to reduce a large number of criteria to a reasonable number to allow reliable and effective risk assessment. The findings of this analysis are presented in Table 4.

### Data Normality Test

The Shapiro–Wilk test examines if a variable is normally distributed in a population. The null hypothesis of the Shapiro–Wilk test is that the data were normally distributed. The test rejects the hypothesis of normality when the *p* value is less than or equal to 0.05, and conclusion that the data are not normally distributed must be made (Royston 1992).

### Intergroup Comparison to Determine Intergroup Statistical Differences

Kruskal–Wallis H test determined whether there were any statically significant differences in respondents’ perception based on their professional roles on the rating of the importance of criteria on identifying the pollutant linkage component. While the *p* value (Asymp. Sig) < 0.05 would reveal a noteworthy difference in the perception of the respondents.

### Intergroups Pair-wise Comparison

Mann–Whitney *U* test is used in this study to perform multiple pair-wise non-parametric comparisons if the Kruskal–Wallis H test shows a significant difference among participants. This test is used to compare differences between two independent groups when the dependent variable is either ordinal or continuous, but not normally distributed (McKnight and Najab 2010).

### Level of Agreement Amongst Participants

In order to check agreements among the participants regarding the ranking of the site criteria to establish pollutant linkage model and the potential hazards associated with brownfield sites, Kendall’s coefficient of concordance (also known as Kendall’s W) test was conducted. Kendall’s W test is a non-parametric statistic. It is a normalisation of the statistic of the Friedman test and can be used for assessing agreement among participants (Rasli 2006). Kendall’s W tests the null hypothesis that “no agreement exists among the rankings given by the participants in a particular group”. It ranges from 0 (no agreement) to 1 (complete agreement) (Lewis and Johnson 1971).

$$W \,=\, 12{\sum} {\frac{{R_i^2 \,-\, 3k^2N\left( {N \,+\, 1} \right)^2}}{{k^2N\left( {N^2 \,-\, 1} \right) \,-\, k{\sum} {T_j} }}}$$

Where: ${\sum} {R_i^2}$ is the sum of the ranks for the individual ranked *N* factors object; *k* is the total number of participants or rankings; and $k{\sum} {T_j}$ is the sum of values of *T*_*j*_ over all *k* sets of ranks.

## Findings

Findings from the analysis of the survey responses are presented and discussed beneath. This section reveals the profiles of the participants (section Demographic Profiles) before analysing and interrogating the data and information returned (section Analysis Findings).

### Demographic Profiles

Following screening of the returned questionnaires and scrutiny for missing data, the final response rate of thirty-eight percent was yielded from 76 complete surveys. The demographic profiles of the survey participants are presented (Table 3). This shows geotechnical and geo-environmental engineers compose most of the participant’s professions (38%; *n* = 29), with hydrologists geochemists, geophysicists and geologists comprising the other roles. Sixty-one percent (*n* = 46) of those taking part in the survey each have more than 6 years’ experience of working as brownfield site professionals.

**Table 3:** Profiles of the participants

Characteristics	Frequency	Percentage (%)
Professions		
Geotechnical engineer	13	17
Geo-Environmental engineer	16	21
Hydrologist	12	16
Geochemist	10	13
Geophysicist	12	16
Geologist	13	17
Years of working experience		
1–3 years	15	20
4–6 years	9	12
More than 6 years	52	68
Years of working experience the development of brownfield sites		
1–3 years	11	14
4–6 years	19	25
More than 6 years	46	61

### Analysis Findings

Before analysing the collected data, the reliability of the data and the normality were tested using the Cronbach’s alpha coefficient test and the Shapiro–Wilk test, accordingly. The calculated Cronbach’s alpha value for the 49 questions was 0.79. This is higher than the threshold of 0.70, which indicates that the measure of the five-point scale and thus the data collected is very reliable for further analysis. Moreover, in this study, all the *p* value calculated by the Shapiro–Wilk test was <0.05, which confirmed that the collected data were not normally distributed. This is expected because for small sample sizes, the sampling distribution of the mean is often non-normal distributed (Royston 1992).

Findings from the analysis of the survey responses are presented and discussed beneath. The results presented in Table 4 reveal that the respondents do not differ based on their roles, only as none of the criteria has its Kruskal–Wallis H test coefficient <0.05, except the ground movement where the results indicated that there is a statistical difference in the perceptions of the six professionals regarding the importance of storage of material and old tank (*X*^2^ = 21.478; *p* value < 0.05; *n* = 76) and invasive species (*X*^2^ = 22.182; *p* value < 0.05 *n* = 76) criteria to determine the ground movement in brownfield sites. Therefore, Mann–Whitney *U* test was conducted to find the cause of the significant differences.

**Table 4:** Summary of the survey results on the criteria for a preliminary risk assessment of brownfield sites (*n* = 76)

	Criteria	Mean	Rank	Kruskal–Wallis H		Kendall’s coefficient of concordance		
				*X*^2^	*P* value	W	*X*^2^	*P* value
Obstruction hazards	Site history	4.74	4	1.376	0.967^a^	0.910^c^	553.556	<0.001
	Surrounding areas	1.05	9	6.059	0.417^a^			
	Building and other structures	4.88	1	3.034	0.804^a^			
	Underground services	4.87	2	5.555	0.475^a^			
	Storage of materials and old tanks	4.84	3	1.333	0.970^a^			
	Previous mining activities	4.72	5	2.944	0.816^a^			
	Presence of radon	1.14	8	1.852	0.933^a^			
	Invasive species	1.15	7	3.681	0.720^a^			
	Made ground	1.51	6	2.324	0.888^a^			
Ground movement	Site history	4.08	3	9.244	0.100^a^	0.816^c^	496.259	<0.001
	Surrounding areas	1.03	8	8.911	0.113^a^			
	Building and other structures	1.36	6	6.640	0.249^a^			
	Underground services	1.30	7	0.843	0.975^a^			
	Storage of materials and old tanks	3.83	4	21.478	0.001^b^			
	Previous mining activities	4.24	2	9.991	0.075^a^			
	Presence of radon	1.02	9	4.857	0.434^a^			
	Invasive species	3.24	5	22.182	0.000^b^			
	Made ground	4.63	1	9.409	0.094^a^			
Chemical contaminants	Site history	4.75	1	6.161	0.405^a^	0.552^c^	335.849	<0.001
	Surrounding areas	4.52	3	3.883	0.693^a^			
	Building and other structures	1.55	9	6.804	0.339^a^			
	Underground services	3.47	8	2.851	0.827^a^			
	Storage of materials and old tanks	4.34	6	6.552	0.364^a^			
	Previous mining activities	4.39	5	7.315	0.293^a^			
	Presence of radon	4.43	1	8.984	0.174^a^			
	Invasive species	3.74	7	6.644	0.355^a^			
	Made ground	4.63	2	1.765	0.940^a^			
Biological hazards	Site history	4.49	2	4.751	0.576^a^	0.823^c^	500.305	<0.001
	Surrounding areas	4.00	4	3.407	0.756^a^			
	Building and other structures	1.42	9	5.155	0.524^a^			
	Underground services	1.47	8	11.101	0.088^a^			
	Storage of materials and old tanks	1.53	6	3.474	0.747^a^			
	Previous mining activities	1.80	5	1.673	0.947^a^			
	Presence of radon	1.51	7	12.349	0.055^a^			
	Invasive species	4.55	1	5.239	0.514^a^			
	Made ground	4.37	3	3.961	0.682^a^			
Biodegradable effects hazards	Site history	4.39	2	5.417	0.367^a^	0.701^c^	426.168	<0.001
	Surrounding areas	3.97	3	4.651	0.460^a^			
	Building and other structures	1.66	6	3.999	0.550^a^			
	Underground services	1.80	4	2.838	0.725^a^			
	Storage of materials and old tanks	1.54	9	10.234	0.069^a^			
	Previous mining activities	1.61	8	6.264	0.281^a^			
	Presence of radon	1.62	7	3.456	0.630^a^			
	Invasive species	1.70	5	5.007	0.415^a^			
	Made ground	4.53	1	3.435	0.633^a^			
Contaminants movement	Site geology (i.e. soil permeability and thickness)	4.64	1	10.214	0.069	0.339^c^	77.354	<0.001
	Site hydrogeology (i.e. presence of groundwater)	3.67	4	1.217	0.943			
	Site hydrology (i.e. presence of surface water and flood zones)	4.53	2	2.927	0.711			
	Site topography (i.e. flat site and steep site)	3.74	3	3.415	0.636			
Receptor	Future user	4.86	1	4.125	0.655	0.457^c^	57.548	<0.001
	Building materials	3.47	2	3.564	0.789			

^a^The Kruskal–Wallis H test result is insignificant at the 0.05 significance level (*p* value > 0.05)

^b^The Kruskal–Wallis H test result is significant at the significance level of 0.05 (*p* value < 0.05)

^c^The Kendall’s W for rating the criteria was W with a significance level <0.001

In addition, Kendall’s W test was performed to calculate the coefficient of concordance. The results of the analysis show a significant degree of agreement exists among all of the participants regarding the ranking of potential hazards associated with brownfield sites.

As mentioned in section 3.5, Mann–Whitney *U* test was used was conducted to find the cause of the significant differences. Starting with the storage of materials and old tanks, the results presented in Table 5 showed that the reason for the statistically significant differences is due to the mean rank of geochemist engineering ($\overline {X_1} \,=\, 8.75$; $\overline {X_2} \,=\, 8.25$; $\overline {X_3} \,=\, 7.75$) were lower than geo-environmental engineering ($\overline {X_1} \,=\, 16.75$), geologist $\left( {\overline {X_2} \,=\, 14.88} \right)$ and geotechnical engineering $\left( {\overline {X_3} \,=\, 15.27} \right)$ respectively. The test indicated that this difference was statistically significant, (*U*_*1*_ = 32.500; *P*_*1*_ = 0.002), (*U*_*2*_ = 27.500; *P*_*2*_ = 0.012), and (*U*_*3*_ = 22.500; *P*_*3*_ = 0.004) successively. In addition, Mann–Whitney *U* test shows that there was significant difference between geophysicists ($\overline {X_4} \,=\, 9.75$; $\overline {X_5} \,=\, 9.58$; $\overline {X_6} \,=\, 8.92$) on the one hand and geo-environmental engineering ($\overline {X_4} \,=\, 18.06$), geologist ($\overline {X_5} \,=\, 16.15$) and geotechnical engineering ($\overline {X_6}$ = 16.77) on the other hand. The test indicated that this difference was statistically significant, (*U*_*4*_ = 39.000; *P*_*4*_ = 0.001), (*U*_*5*_ = 37.000; *P*_*5*_ = 0.017), and (*U*_*6*_ = 29.000; *P*_*6*_ = 0.004) successively. Mann–Whitney *U* test shows also that was significant difference between hydrologists ($\overline {X_7} \,=\, 10.96$; $\overline {X_8} \,=\, 9.67$) and geo-environmental engineering ($\overline {X_7} \,=\, 17.16$) and geotechnical engineering ($\overline {X_8} \,=\, 16.08$). The test marked that this difference was statistically significant, (*U*_*7*_ = 53.500; *P*_*7*_ = 0.017) and (*U*_*8*_ = 38.000; *P*_*8*_ = 0.019) successively.

**Table 5:** Significant differences for storage of materials and old tanks (*n* = 76)

N°	Job category	Mean rank	Mann–Whitney *U* test		
			U	Z	*P* value
1	Geochemist	8.75	32.500	−3.080	0.002
	Geo-Environmental engineering	16.47			
2	Geochemist	8.25	27.500	−2.502	0.012
	Geologist	14.88			
3	Geochemist	7.75	22.500	−2.848	0.004
	Geotechnical	15.27			
4	Geophysicist	9.75	39.000	−3.200	0.001
	Geo-Environmental engineering	18.06			
5	Geophysicist	9.58	37.000	−2.387	0.017
	Geologist	16.15			
6	Geophysicist	8.92	29.000	−2.856	0.004
	Geotechnical	16.77			
7	Hydrologist	10.96	53.500	−2.386	0.017
	Geo-Environmental engineering	17.16			
8	Hydrologist	9.67	38.000	−2.339	0.019
	Geotechnical	16.08			

Regarding invasive species criteria, Mann–Whitney *U* test was applied to find the cause of the significant differences, the results are presented in Table 6. The results show that the reason for the statistically significant differences is due to the mean rank of geophysicists ($\overline {X_1} \,=\, 10.42$, $\overline {X_2} \,=\, 8.71$; $\overline {X_3} \,=\, 8.75$) were lower than geo-environmental engineering ($\overline {X_1} \,=\, 17.56$) geotechnical engineering, ($\overline {X_2} \,=\, 16.96$) and geologist ($\overline {X_3} \,=\, 16.92$) respectively. The test indicated that this difference was statistically significant, (*U*_*1*_ = 47.000; *P*_*1*_ = 0.018), (*U*_*2*_ = 26.500, *P*_*2*_ = 0.003) and (*U*_*3*_ = 27.000; *P*_*3*_ = 0.004) successively. Furthermore, Mann–Whitney *U* test shows that there was significant difference between hydrologist ($\overline {X_4} \,=\, 10.33$; $\overline {X_5} \,=\, 8.33$ and $\overline {X_6} \,=\, 8.50$) and geo-environmental engineering ($\overline {X_4} \,=\, 17.63$), geotechnical engineering ($\overline {X_6}$ = 17.31) and geologist ($\overline {X_5} \,=\, 17.15$). The test indicated that this difference was statistically significant, (*U*_*4*_ = 46.000; *P*_*4*_ = 0.015), (*U*_*5*_ = 22.000; *P*_*5*_ = 0.001), and (*U*_*6*_ = 24.000, *P*_*6*_ = 0.002) successively. Mann–Whitney *U* test shows also that was significant difference between geochemist ($\overline {X_7} \,=\, 8.50$; $\overline {X_8} \,=\, 8.60$) on the one hand and geotechnical engineering ($\overline {X_7} \,=\, 14.69$) and geologist ($\overline {X_8} \,=\, 14.62$) on the other hand. The test marked that this difference was statistically significant, (*U*_*7*_ = 30.000; *P*_*7*_ = 0.013) and (*U*_*8*_ = 31.000; *P*_*8*_ = 0.025) successively.

**Table 6:** Significant differences for invasive species (*n* = 76)

N°	Job category	Mean rank	Mann–Whitney		
			U	Z	*P* value
1	Geophysicist	10.42	47.000	−2.356	0.018
	Geo-Environmental engineering	17.56			
2	Geophysicist	8.71	26.500	−2.990	0.003
	Geotechnical	16.96			
3	Geophysicist	8.75	27.000	−2.873	0.004
	Geologist	16.92			
4	Hydrologist	10.33	46.000	−2.422	0.015
	Geo-Environmental engineering	17.63			
5	Hydrologist	8.33	22.000	−3.305	0.001
	Geotechnical	17.31			
6	Hydrologist	8.50	24.000	−3065	0.002
	Geologist	17.15			
7	Geochemist	8.50	30.000	−2.475	0.013
	Geotechnical	14.69			
8	Geochemist	8.60	31.000	−2.245	0.025
	Geologist	14.62			

This study also analysed the participants’ agreement regarding the ranking of potential hazards associated with brownfield sites. Kendall’s W test result of W with the small associated level of significance of 0.001 (*n* = 76) implied that there was a significant degree of agreement between the respondents regarding the necessary criteria to identify hazards in brownfield sites. This signifies that there is a strong agreement among the six professionals of participants on the importance rating of criteria to determine the pollutant linkage components. The outcome of this analysis is presented in Table 4.

## Discussion

Based on the design of the source–pathway–receptor model, this section discusses the findings and then considers the potential issues and implications.

### Source—Obstruction Hazards

By previous use, brownfield sites contain buildings, ancillary structures, and underground services. These pose potential barriers to redevelopment, which could be of great consequence if not anticipated and planned when discovered during construction (Barry 1991). The results show a significant degree of agreement between the professionals regarding the necessary criteria to identify obstruction hazards in brownfield sites. Six professional groups agreed on the importance rating of criteria to determine the potential obstructions.

In general, buildings and other structures (mean = 4.88; SD = 0.325; *n* = 76) were perceived to be the most important criteria to identify obstruction in brownfield sites. This is expected result as it is common to find obstruction in brownfield sites. Moreover, underground service criteria is essential because damage to underground services can cause fatal or severe injury. For example, underground electrical cables carry considerable hazardous because they often look like pipes and it is hard to know if they are live just by looking at them. This criteria was rated extremely important by mean = 4.87 (SD = 0.340; *n* = 76). As expected, storage of materials and old tanks was rated high by mean = 4.84 (SD = 0.367; *n* = 76) amongst the criteria to identify obstructions in brownfield sites, mainly because they present a potential obstruction to redevelopment which, if not foreseen and planned for, can have a major significance when discovered during construction. History of the site rated with mean = 4.74 (SD = 4.74; *n* = 76), which provides evidence that this criteria is emphasised by the experts, as an extremely important indication of potential obstruction. Participants perceive “Previous mining activities” (mean = 4.72; SD = 0.532; *n* = 76) criteria as vital to identify obstruction (i.e. underground pipe runs, tanks, etc.). This finding is consistent with the previous study by Leach and Goodger (1991) concerning the physical hazards in derelict sites.

### Source—Ground Movement

Brownfield sites have the potential for ground movement, where settlement is the most common form but, in certain situations, the ground may heave (Charles 2005). The findings show that made ground was ranked first by professionals (mean = 4.63; SD = 0.608; *n* = 76). This result is in great agreement with studies (Watts and Charles 1997; Charles and Skinner 2004) showed a significant issue to the foundations of buildings due to the compressibility of the ground. Criteria related to previous mining activities ranked second by mean = 4.24 (SD = 0.781; *n* = 76). It is understandable because such an industry may leave a wide amount of slags that cause expansion on wetting (Charles et al. 2002). The third issue ranked by participants was criteria related to the history of the site (mean = 4.08; SD = 0.648; *n* = 76). These findings were highlighted by a study conducted by Sivapullaiah et al. (2009) who demonstrated that the swelling of soil in the presence of waste material such as sulfuric acid is highly likely due to the leaching of fixed potassium ions from between the interlayers. Storage of materials and old tanks criteria ranked fourth by mean = 3.83 (SD = 0.915; *n* = 76), although geophysicist, geochemists, and hydrologist do not rank this criteria important to identify ground movement, it was ranked extremely important by other professionals as it raises concerns about the ground instability related to removing tanks and underground storages as highlighted by previous study by Barry (1991).

Although the invasive species (mean = 3.38; SD = 0.821; *n* = 76) was <3.40, it was marginally important as a number of professionals including geo-environmental engineers, geotechnical engineers, and geologists considered invasive species as important criteria to identify the ground movement in brownfield sites, where they are known to cause significant landslides and soil loss in areas that are colonised by Himalayan balsam (Greenwood and Kuhn 2014). This hazard was underestimated by geophysicists, hydrologists, and geochemists the importance of this criteria to identify ground movement.

### Source—Chemical Hazards

Chemicals are one of the most important hazards arising from industrial use, which may present a major threat to humans. History of the site criteria provides a good indication of potential sources and types of chemicals likely to be found on site. As expected, participants ranked first this criteria as extremely important by mean = 4.75 (SD = 0.465; *n* = 76). The second, as the participants ranked was made ground by mean = 4.63 (SD = 0.538; *n* = 76). This expected as made ground may cause pollution, where liquid waste (Leachate) leaking is a major issue related to ground pollution (Sarsby and Felton 2006). Surrounding area criteria was ranked third with a mean = 4.52 (SD = 0.608; *n* = 76). This is expected, mainly because, in areas where the surrounding sites are known by historical industrial activities, it can be considered as a source of contamination, because the behaviour of the site containing contamination is the long-term migration of the contaminants itself to potential receptors (Gurunadha Rao and Gupta 2000). The criteria related to the presence of radon ranked fourth by participants with a mean = 4.43 (SD = 0.736; *n* = 76) as it is the most common source of exposure to radiation, easily exceeding exposure from nuclear power stations or hospital scans and X-rays (EPA 2019). Previous mining activities criteria was ranked fifth by mean = 4.39. This can be explained as such as criteria is a good indicator to identify a range of chemical contaminants in particular steel-making processes (Charles 2005). Storage of materials and old tanks was ranked sixth by mean = 4.34. This finding is consistent with the previous study by Motta et al. (2017), and Beiras (2018) concerning the fuel storage and distribution at industry manufacture as one of the main causes of soil and groundwater contamination, due to leakage from piping, from underground storage tanks. The criteria related to invasive species ranked seventh by mean = 3.74 (SD = 0.943; *n* = 76). According to Elliott (2003), this criteria can help investigators to identify chemical hazards that may cause serious health issues including poisoning, scars, and blindness if the sap gets into the eyes. The results (Table 4) indicated that there is not statistically different in the perceptions of the six professionals, as none of the criteria has its Kruskal–Wallis H test coefficient <0.05.

### Source—Biological Contaminants

There are a number of biological hazards that may be exist on a brownfield site and any of these could lead to disease if precautions are not taken to reduce the risks. Some of these diseases can be serious or fatal (Kovacs and Szemmelveisz 2017). It is not surprising that the history of the site ranked first by mean = 4.55 (SD = 0.501; *n* = 76) because industries and activities such as sewage, hospital waste, landfills, canals, laboratory waste and disease/burial pits are the main sources for bacteria, fungi, parasites and viruses. Made ground ranked second by mean = 4.49 (SD = 0.663; *n* = 76). This can be explained as wastes contaminated with biological materials could lead to disease if precautions are not taken to reduce the risks. Thirdly, surrounding areas by mean = 4.37 (SD = 0.538; *n* = 76). This criteria is extremely useful because surrounding areas are known by industrial activities, it can be considered as a source of biological contamination, which may migrate to potential receptors. Although the results confirmed the similarity in the perception of professionals about the most appropriate criteria to identify the biological contaminants in brownfield sites, invasive species criteria was underestimated by most of the participants and this contradicts a study conducted by (Elliott 2003) which considered invasive species as biological pollution were, the terms biological pollutants have been used by (Boudouresque and Verlaque 2002) to discuss the problems caused by such invasive species. Therefore, there is a need to enhance the knowledge of professionals concerning the biological hazards of invasive species.

### Source—Biodegradable Hazards

Participants ranked made ground first by mean = 4.53 (SD = 0.663; *n* = 76) to identify biodegradable effects in brownfield sites. This criteria provides a good indicator about the hazards related to biodegradable materials during the long process of decomposition, where biological reactions in landfills can convert organic compounds to several different gases, called biogas Talaiekhozani et al. (2018). In addition, the history of the site was rated also extremely important because it generally provides a good indication of former waste disposal sites that contain biodegradable materials. These criteria ranked second by mean = 4.42 (SD = 0.634; *n* = 76). Surrounding areas criteria ranked third by mean = 3.97 (SD = 0.588; *n* = 76). This finding was highlighted by many studies (Kanmani and Gandhimathi 2013; Locatelli et al. 2019), where the accumulation of landfill gas may attribute to lateral migration of landfill gas from old waste fill sites to adjacent sites. Landfills gas can migrate significant distances because it is affected particularly by ground permeability. The results presented in Table 4 confirmed that the individual groups did not differ significantly, as none of the criteria has its Kruskal–Wallis H test coefficient <0.05.

### Pathway—Contaminants Movement

Pathway identifies how hazards were released from the source into the environment (Butt et al. 2016). It is mainly subjected to investigate the impact of site conditions on the fate and transport of contaminants (Wu et al. 2019). Criteria related to site geology (i.e. soil permeability and thickness) ranked first by mean = 4.64 (SD = 0.559; *n* = 76). This can be explained as soil permeability parameter is one of the most important factors within the pathway process where contaminant movement is more likely in a highly permeable layer than an impermeable layer. In addition, the soil thickness parameter also plays an essential role when assessing contaminants pathway movement, as the thicker the layer the longer takes the contaminants to move through it (British Standard 2015). Site hydrology (i.e. presence of surface water and flood zones) ranked second by mean = 4.53 (SD = 0.589; *n* = 76), this criteria plays also a critical role when assessing possible pathways because it influences the movement of potential contaminants and the potential exposure pathways to human health and environmental receptors. While site topography (i.e. flat site and steep site) ranked third by mean = 3.74 (SD = 0.737; *n* = 76). It is understandable why this criteria ranked important by participants because it plays an important role in identifying the direction of the contaminant pathway. Site hydrogeology (i.e. presence of groundwater) ranked fourth by mean = 3.67 (SD = 0.90; *n* = 76). This criteria provides a useful reminder to assessors that the presence of groundwater and/or surface water assists the movement of contaminants, therefore increasing the risk of contaminants migration.

It can be seen that all criteria does not show a significant difference between job categories. This signifies that there is a strong agreement among the six professionals of participants on the importance rating of criteria to determine the potential obstructions.

### Receptors—Future Land Users and Building Materials

Risks posed to human health is usually the dominant issue in the redevelopment of brownfield sites (Skinner et al. 2005). It is expected that future end-use criteria ranked extremely important to identify hazards posed to human health by mean = 4.86 (SD = 0.896; *n* = 76). Otherwise, criteria related building materials considered important by mean = 3.47 (SD = 0.768; *n* = 76) to assess the risks posed to buildings because at brownfield sites, building materials are often subjected to aggressive environments that cause them to physical or chemical changes. The results show that there is a strong agreement among the six professionals of participants on the importance of criteria related to the future user and building materials to determine the potential targets.

## Potential Issues and Implications

The starting point of the brownfield risk assessment process is hazard identification, which is a complex relationship of sources, pathways and receptors (Environment Agency 2008). This process is often quite time consuming as it usually involves gathering a vast number of criteria to fully assess a potentially hazards. Therefore, there is a need for toolkit/mechanism of appropriate criteria which assist specifically in connection to contaminated sites for clearing and redevelopment via land reclamation. Such a toolkit is to save time, effort and other resources. essential that the correct criteria required for the development of such a site is collected and used in the most cost-effective manner.

This paper produces a set of criteria to assist in identifying the possibility of existence of hazards in a given brownfield/contaminated site. This process is not to capture the degree of ‘hazardousness’/concertation of a hazard as an whether it is below or above an acceptable safe level of concertation. The idea is to save risk assessors and other associated stakeholders from investing their time, effort, cost and other resources in the hunt of those hazards which are not possible to exist in the first place. For instance, the history (which one of the criteria) of a brownfield site is oil abstraction or petrol station, then the risk assessor focus should be to establish the existence of hydrocarbons in the soil regardless of the degree of concentration of hydrocarbons, be it lower or higher than the safety levels for a given scenario. Furthermore, another criterion is regarding the sensitivity of the potential receptor. If, continuing from the same example, a school is to be built or playground for children then the process would indicate the direction and the depth of the follow-on detailed risk assessment, as appropriate. On the other, if a car park is constructed then that would accordingly reduce the depth of the follow-on risk assessment exercise. In summary, the criteria identified in this study time and cost effectively set the scene for follow-on measures in terms of amount, depth and direction.

This study reveals challenges facing the investigators of brownfield sites to identify the risks and hazards associated with brownfield site development. The risk assessment process is sometimes failed by assessors where many of application were refused by local authorities due to not comprehensively and successfully identify potential hazards. Another challenge in the assessment of brownfield sites is commonly required expertise and knowledge from a number of disciplines, ranging from geotechnical engineers to geochemical scientist to provide an independent professional report about the risks, particularly to human health and the built environment, by identifying actual or potential hazards of the site (Nathanail and Bardos 2005; Nathanail et al. 2011). According to the Environment Agency (2008), the lack of criteria increases uncertainties in identifying and assessing hazards, which leads to poor communication between stakeholders, possibly leading to different suitably qualified stakeholders reaching to different conclusions even when presented with the same criteria. However, excessive detail should be avoided, and the level of detail should be no more than is needed for robust decisions to be taken.

The findings of this study clarify both the key criteria requirements for the preliminary risk assessment of brownfield sites, as well as the importance of recognising how variation in professionals’ perceptions plays into the risk assessment process. Even though specialist knowledge is fundamental to the brownfield investigation, maintaining a wide perspective of experts coming from different backgrounds is critical, as this makes the risk assessment more comprehensive and complete. This encourages the reuse of brownfield sites, especially in countries that have adopted a policy of preservation of green fields and enhancing sustainable redevelopment.

**Fig. 2:**
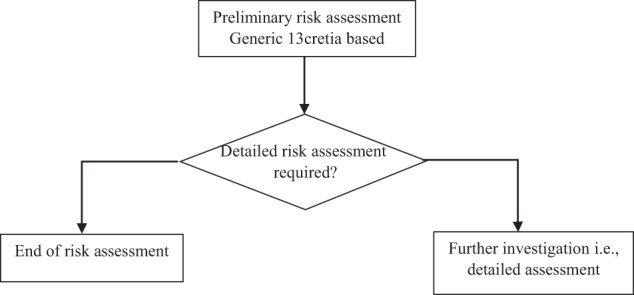
Preliminary risk assessment of brownfield site 13 criteria based

The identified generic criteria are for preliminary risk assessment stage to be a cost effective. However, when the outcome of the preliminary risk assessment suggests carrying out a detailed risk assessment, at that point these generic criteria can be investigated in lot more site-specific context for a given brownfield site. Figure 2 shows preliminary risk assessment (PRA) model 13 criteria based. The criteria for the initial risk assessment will depend on the context and objectives of the risk assessment, as well as on the general characteristics of the site. The criteria provide an indication of the general type of information that may be required for an initial risk assessment. The evaluator will need to identify the specific information required in any situation and focus the information gathering on meeting those information needs.

## Conclusions and Future Research

This study aimed to determine the criteria necessary for the initial risk assessment of brownfield site based on the pollutant linkage model (Source–Pathway–Receptor) with focus on the level of agreement and disagreement between expert groups in their perception of the criteria requirements. A total of thirteen criteria were identified through a systematic review and presented to expert groups to gauge their level of importance in relation to preliminary assessment of brownfield sites. Participants were required to identify the appropriate criteria to identify the pollutant linkage components.

The results of statistical analyses of seventy-six expert responses indicate that the top criteria to identify the source of hazards are history of the site, made ground, invasive species, previous mining, storage of materials and old tanks, presence of radon, underground services and buildings and other structures. Furthermore, site geology, site hydrology, site hydrogeology and site topography were rated as the top criteria to identify the pathway movement of the contaminants. While future site use scenario criteria is critical to identify the critical receptor of the population most likely to be exposed and/or susceptible to the presence of soil contamination.

The study renders the preliminary risk assessment exercise to be not only more holistic and integrated but also to reduce uncertainty in risk assessment by ensuring that all eventualities along with their respective significance have been encapsulated at the initial stage of risk assessment. Another important element of the study brought out is that the same hazard and associated risk can be of varying significance to different professionals. So much so that a crucial hazard in the eyes of one practitioner may not be a hazard at all in the eyes of another practitioner, merely due to the difference in their backgrounds. This variation in views and interests of different professionals can help the risk assessor to develop the pollutant linkage model of the brownfield site more categorically and systemically, encapsulating all possible hazards, pathways and receptors. A diversity of professional engagements would enhance the capability of the risk assessor to signify and appropriately prioritise hazards in the preliminary risk assessment with greater confidence.

Finally, this study advocates the need for more inclusive criteria to come from the perspective of various professional practitioners in view of their different backgrounds; thereby, enabling more holistic and complete identification of hazards (with their diverse implications) for a given brownfield site.

Based on the findings revealed in this study the following recommendations are proposed:

1. Future research could also determine the total population of professionals in the brownfield redevelopment sector and employ a larger sample to comprehensively analyse the differences between professionals’ perceptions.
2. Lastly, future research could attempt validate the findings of this study through real case studies of risk assessment to quantify and show the real benefits to policy makers, industry stakeholders, which could make preliminary risk assessment of brownfield sites more attractive for them.
3. The idea of carrying out a PRA prior to detailed risk assessment (which is more costly and time consuming and a liber intensive) can be enhanced by developing a full-on model and validated via applying to wide range of brownfield site.
